# Argonaute2 Suppresses *Drosophila* Fragile X Expression Preventing Neurogenesis and Oogenesis Defects

**DOI:** 10.1371/journal.pone.0007618

**Published:** 2009-10-27

**Authors:** Anita S.-R. Pepper, Rebecca W. Beerman, Balpreet Bhogal, Thomas A. Jongens

**Affiliations:** Department of Genetics, University of Pennsylvania School of Medicine, Philadelphia, Pennsylvania, United States of America; Columbia University, United States of America

## Abstract

Fragile X Syndrome is caused by the silencing of the Fragile X Mental Retardation gene (FMR1). Regulating dosage of *FMR1* levels is critical for proper development and function of the nervous system and germ line, but the pathways responsible for maintaining normal expression levels are less clearly defined. Loss of *Drosophila* Fragile X protein (dFMR1) causes several behavioral and developmental defects in the fly, many of which are analogous to those seen in Fragile X patients. Over-expression of dFMR1 also causes specific neuronal and behavioral abnormalities. We have found that Argonaute2 (Ago2), the core component of the small interfering RNA (siRNA) pathway, regulates *dfmr1* expression. Previously, the relationship between dFMR1 and Ago2 was defined by their physical interaction and co-regulation of downstream targets. We have found that Ago2 and dFMR1 are also connected through a regulatory relationship. Ago2 mediated repression of dFMR1 prevents axon growth and branching defects of the *Drosophila* neuromuscular junction (NMJ). Consequently, the neurogenesis defects in larvae mutant for both *dfmr1* and *Ago2* mirror those in *dfmr1* null mutants. The *Ago2* null phenotype at the NMJ is rescued in animals carrying an *Ago2* genomic rescue construct. However, animals carrying a mutant *Ago2* allele that produces Ago2 with significantly reduced endoribonuclease catalytic activity are normal with respect to the NMJ phenotypes examined. dFMR1 regulation by *Ago2* is also observed in the germ line causing a multiple oocyte in a single egg chamber mutant phenotype. We have identified Ago2 as a regulator of *dfmr1* expression and have clarified an important developmental role for Ago2 in the nervous system and germ line that requires *dfmr1* function.

## Introduction

Fragile X mental retardation syndrome is the most common heritable form of mental retardation and known cause of autism. In mammals, the dosage of Fragile X expression is critical to the distinct diseases related to this locus. In most patients with Fragile X syndrome, the *FMR1* gene is transcriptionally silenced when the CGG triplet repeat in the 5′-untranslated region (UTR) is methylated upon expansion to greater than 200 copies [1], [2], [3]. Over-expression of the CGG containing *FMR1* transcript itself is linked to both an independent neurodegenerative disease, Fragile X-associated tremor/ataxia syndrome (FXTAS) and to premature ovarian failure [4], [5], [6], [7], [8], [9]. It is not fully understood how the steady state levels of the *FMR1* gene are normally regulated; however, it is clear that tight regulation, both positive and negative, is required for proper neuronal and germ-line function and maintenance (Reviewed in [10]).

The *Drosophila melanogaster* Fragile X model, based on the single *Drosophila fragile X mental retardation gene* (*dfmr1*), has proven itself a facile system for understanding aspects of the genetic, molecular, cognitive and morphological defects that affect Fragile X and FXTAS patients (Reviewed in [11]). dFMR1 shares extensive homology with human FMR1 in the RNA binding motifs: the K homology (KH) domains and the RGG-type RNA-binding domain [12], [13], [14], [15]. Similar to *FMR1*, *dfmr1* mRNA is expressed throughout development with the highest levels seen in neurons and the *Drosophila* germ line [15], [16], [17]. Like its human counterpart, regulation of *dfmr1* is required for normal neuronal development and function. Over-expression of FMR1 in a mouse model system has been shown to result in abnormal behavioral and neurological activities [18], [19]. Analogously, over-expression of dFMR1 in the brain causes behavioral defects, axon guidance and extension defects, and dendritic branching abnormalities [20], [21], [22], [23], [24]. Both the human and *Drosophila* fragile X proteins bind their own mRNA and are involved in translational regulation [25], [26], [27]. Interestingly, the dFMR1 protein has been shown to act as both a negative regulator of target transcripts, such as *Futsch* and *pickpocket*, and a positive regulator of target transcripts, such as *Trailer Hitch* [27], [28], [29].

Although dFMR1 is hypothesized to act as a translational regulator, no single mechanism has emerged to explain how dFMR1 regulates its targets. One potential mechanism for how dFMR1 could function as a translational regulator is based on the physical association between dFMR1 and Argonaute2 (Ago2), the core component of the small interfering RNA (siRNA) pathway [29], [30], [31]. Specifically, Ago2 functions as an endoribonuclease in a protein complex bound to short RNAs that serve as guides to target specific transcripts for degradation (Reviewed in [32]). Because dFMR1 was found to be present in the functional siRNA induced silencing complex (siRISC) with Ago2, it was hypothesized that this multi-protein complex could mediate dFMR1-associated translational regulation. Additionally Ago2 and dFMR1 were also shown to co-regulate a target mRNA suggesting that these two proteins function coordinately[29]. However unlike Ago2, which is required for siRNA mediated silencing, dFMR1 has been shown to have little to no effect on siRNA efficiency, and is therefore thought to play a stabilizing or modifying role in siRISC function [29], [30], [31].

Ago2 has been well characterized as the core component of the siRNA pathway and the endogenous siRNA pathway, yet fewer studies have examined the biological role of Ago2 in animal models. Ago2 has been shown to be important in larval behavior and proper embryogenesis but the pathways and mechanisms by which these Ago2 mediated processes occur are unknown [29], [33], [34].

The aim of our research was to determine how Ago2 and dFMR1 interact genetically in two developmental systems known to be dependent on dFMR1 expression, the neuromuscular junction and the female germ line. By looking at dFMR1 expression in a variety of genetic backgrounds and in different tissue types, we have found that Ago2 regulation of dFMR1 in the nervous system and the germ line is necessary for proper neurogenesis and oogenesis.

## Results

### Loss of Ago2 causes defects in synaptic structure that are dependent on *dfmr1*

**Figure 1:**
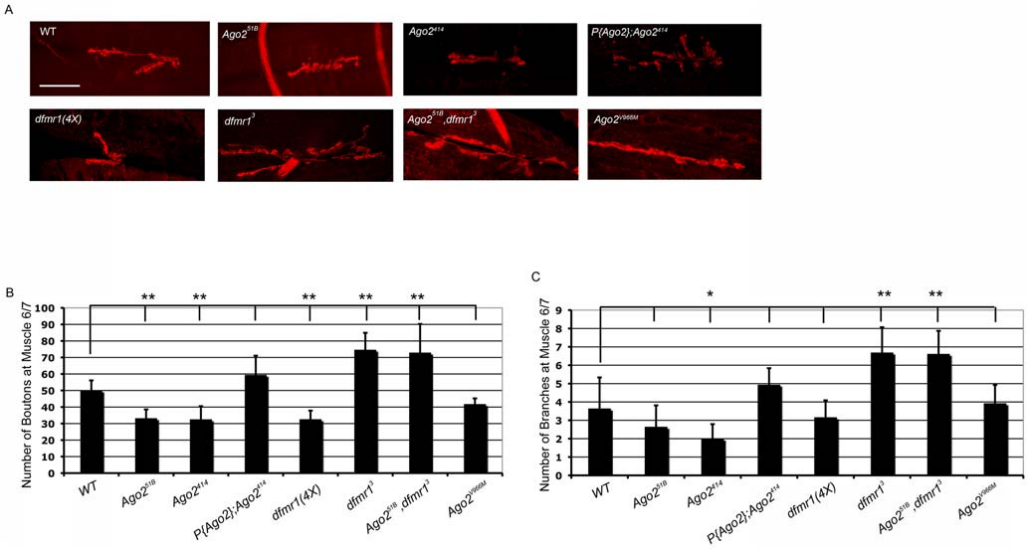
Ago2 affects synaptic development and morphology similarly to dFMR1 over-expression. (A) Representative images show third instar larval, abdominal hemisegment 3, muscle 6/7 of the NMJ marked with Texas-Red conjugated HRP. *dfmr1(4X)*, *Ago2^414^*, and *Ago2^51B^* NMJs show under-elaborated branching, whereas *dfmr1* null and *Ago2^51B^, dfmr1^3^* double mutants show over-elaboration of branching patterns, and *P{Ago2};Ago2^414^ and Ago2^V966M^* are similar to WT. Scale bar is 41.66 µm for WT, *dfmr1(4X), dfmr1^3^, Ago2^51B^*, and *Ago2^51B^, dfmr1^3^*, and *Ago2^V966M^* (the *Ago2^V966M^* allele is presented here although its significance is discussed later in the results section). Scale bar is 50 µm for *P{Ago2};Ago2^414^*, and *Ago2^414^*. (B and C) Quantification of structural phenotypes at the synapse. Numbers of type I synaptic boutons (B) and numbers of type I synaptic branches (C) are quantified for abdominal hemisegment 3, muscle 6/7 of the NMJ. For all genotypes, n≥13. *P<0.001 and **P<0.0001 using one-way ANOVA followed by Dunnett's test. Data are graphed as mean ± s.d.

It was previously shown that dFMR1 is expressed in the pre-synaptic motor neurons and post-synaptic muscles in larvae [27]. Effects from changes in dFMR1 levels have been well characterized in the larval neuromuscular junction (NMJ), which is an excellent model to study synaptic structure [27]. *dfmr1* null animals have over-elaborated synaptic termini with an increased number of smaller synaptic boutons. In contrast, the over-expression of dFMR1 leads to a decreased number of larger synaptic boutons and a loss of synaptic branching. For consistency we carried out all of our studies of the NMJ in the same abdominal hemisegment (3), and muscle (6/7) in third instar wandering larvae. The bouton number and branch numbers of the synaptic termini were quantified using anti-cysteine string protein and DAB staining visualized with a light microscope. Additionally we used anti-HRP to observe the gross morphology of the larval NMJ architecture of the same abdominal hemisegment (3), and muscle (6/7) using a maximum projection from a stack of confocal sections through the NMJ (Figure 1A).

The larval NMJ from two independently isolated *Ago2* null strains, *Ago2^51B^* [29] and *Ago2^414^* [35], exhibited a significant under-elaboration of the synapse with a 34% decrease in the number of boutons in comparison to the wild-type larval NMJ (WT) (Figure 1). To verify that the NMJ defects observed in the *Ago2* null mutants were due to loss of Ago2 activity and not genetic background, we also analyzed larvae carrying a genomic *Ago2* transgene in an *Ago2* null background, *P{Ago2}; Ago2^414^* larvae [35]. Both the bouton and branching phenotypes observed in *Ago2* null larvae were rescued in the *P{Ago2};Ago2^414^* larvae. The NMJ phenotype observed in *Ago2* null larvae was strikingly similar to that observed in larvae where dFMR1 is over-expressed using the UAS/GAL4 system [27]. Similarly, transgenic larvae carrying four copies of *dfmr1*, *dfmr1(4X)* also displayed under-elaboration of the synapse with a 36% decrease in the number of boutons in comparison to WT (Figure 1).

To determine whether dFMR1 was required for the *Ago2* null NMJ phenotype, we carried out genetic epistasis analyses. The *dfmr1* null (*dfmr1^3^*) larvae displayed an opposite NMJ phenotype in comparison to the larvae that over-express *dfmr1*. Loss of *dfmr1* resulted in over-elaboration of the synaptic termini with a 48% increase in the number of boutons and 81% increase in the number of synaptic branches in comparison to WT, similar to the phenotypes observed in the mammalian nervous system of *FMR1* mutants and Fragile X patients (Figure 1) [36], [37], [38], [39]. If dFMR1 over-expression were required for the *Ago2* null phenotype, then we would expect that *Ago2^51B^, dfmr1^3^* double mutant animals would resemble *dfmr1* ^3^ animals. *Ago2^51B^, dfmr1^3^* double mutant larvae did indeed display a *dfmr1* null-like phenotype with respect to the gross morphology of the NMJ. Specifically, we observed a 45% increase in the bouton numbers from *Ago2^51B^, dfmr1^3^* double mutant larvae compared to wild-type *larvae*, and an 87% increase in synaptic branch numbers (Figure 1). We therefore were able to conclude that loss of Ago2 expression alters the larval NMJ synaptic structure through dFMR1.

### Ago2 regulates dFMR1 in the adult nervous system

**Figure 2:**
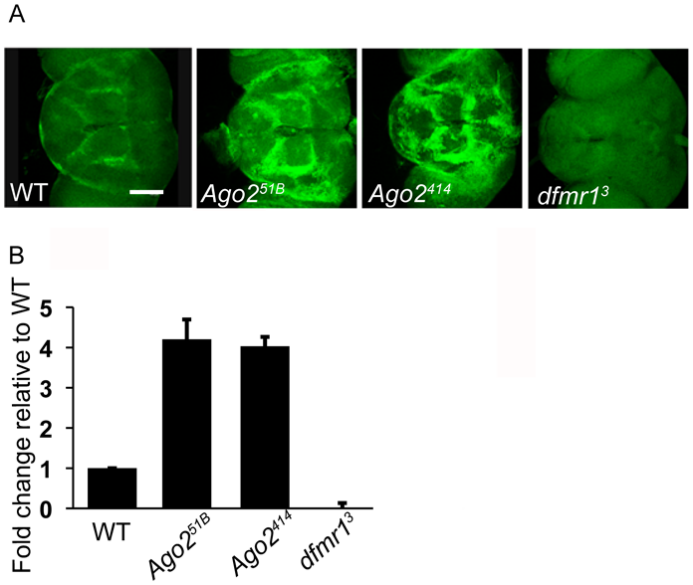
Loss of Ago2 results in increased dFMR1 in adult heads. (A) Representative whole-mount brains labeled for dFMR1 (green) from WT, *Ago2^51B^*, *Ago2^414,^* and *dfmr1^3^* flies. Scale bar is 100 µm. (B) Graphical representation of fold increase in fluorescence (representing dFMR1) intensity relative to WT as quantified by Leica TCS SP quantification software (n≥5 for each genetic background). See Materials and Methods for details. Data are graphed as mean ± s.d.

To determine whether the larval NMJ phenotype observed in *Ago2* null larvae was actually due to over-expression of dFMR1 as was suggested by the genetic studies, we examined how loss of Ago2 affected dFMR1 expression in adult brains. Immunohistochemistry analyses in whole-mount brains revealed that the expression of dFMR1 is more than four-fold higher in brains from *Ago2^51B^* and *Ago2^414^* flies compared to expression in wild-type brains (Figure 2). Loss of Ago2 did not affect the spatial patterns of dFMR1 expression in the adult brain. In addition, no gross morphological mutant phenotypes were observed in the *Ago2* null fly brains (Figure 2). Western analysis of whole head extracts prepared from control and *Ago2* null flies also showed similar up-regulation of dFMR1 protein levels (Figure S1A, B).

### Ago2 regulates dFMR1 during oogenesis

**Figure 3:**
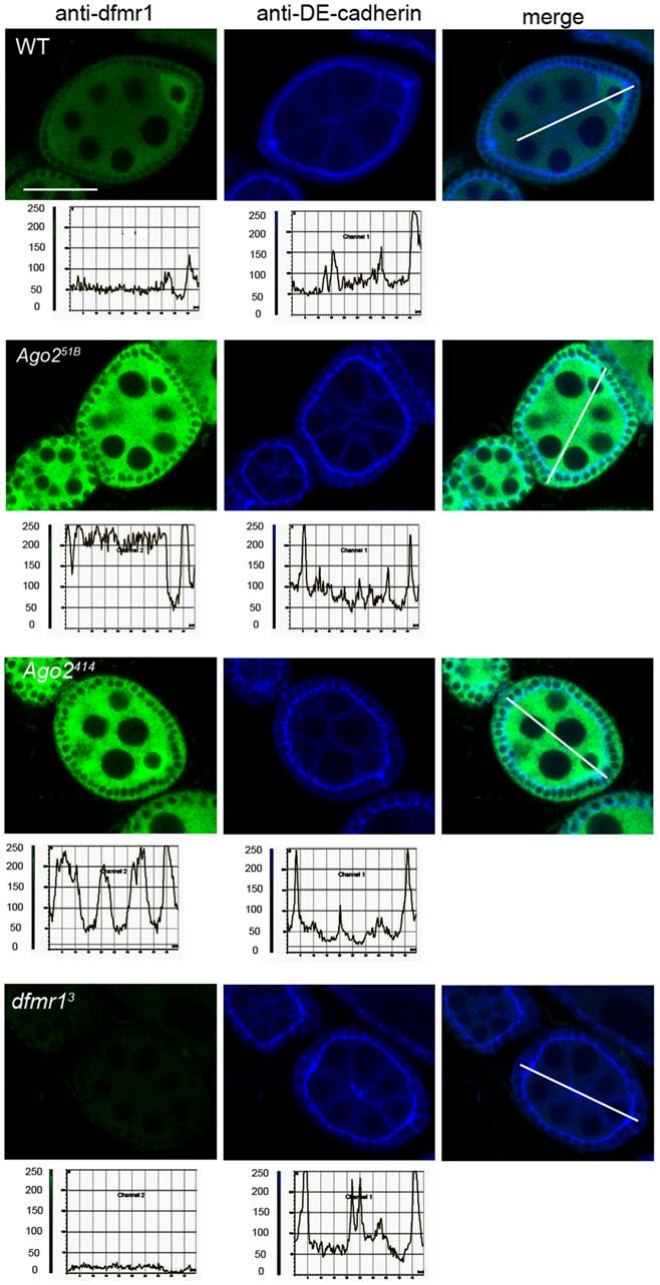
Loss of Ago2 results in increased dFMR1 in oogenesis. Representative images of whole-mount ovaries labeled for dFMR1 (green) and DE-cadherin (blue) from WT, *Ago2^51B^*, *Ago2^414^*, and *dfmr1^3^* flies. Scale bar is 28.63 µm. The graphs plot the pixel intensity (0 to 255 as quantified by Leica software) versus position (µm) along a line drawn through the egg chamber (seen in the merged image) (n≥10 for each genetic background). See Materials and Methods for details.

Previously we had observed that tight regulation of dFMR1 was required in the female germ line for proper development, therefore we asked whether Ago2 regulated dFMR1 in other non-neuronal tissues such as the ovaries. We analyzed dFMR1 expression throughout oogenesis using immunofluorescence staining on ovaries from both *Ago2* null strains. dFMR1 is normally enriched in the oocyte but present throughout the egg chamber at low levels [40]. The ovaries from flies lacking Ago2, maintained the wild-type enrichment of dFMR1 in the oocyte (Figure 3 and [40]). Paralleling results from adult brains, quantitative comparisons of dFMR1 expression levels in ovaries from both *Ago2* mutants and WT ovaries revealed up-regulation between 2–4 fold of dFMR1 protein levels throughout the *Ago2* null egg chambers (Figure 3). Additionally, levels of DE-cadherin, a cell adhesion molecule, were consistent in egg chambers from all genotypes, substantiating the uniformity of the staining technique. Co-staining with anti-dFMR1 and anti-DE-cadherin also demonstrated that the up-regulation of dFMR1 observed in *Ago2* null egg chambers was likely specific and not due to global regulation of translation or protein stability by Ago2. Western analyses of extracts prepared from control and *Ago2* null ovaries also revealed similar up-regulation of dFMR1 protein levels (Figure S1C, D).

### Loss of Ago2 causes oogenesis defects similar to dFMR1 over-expression

**Figure 4:**
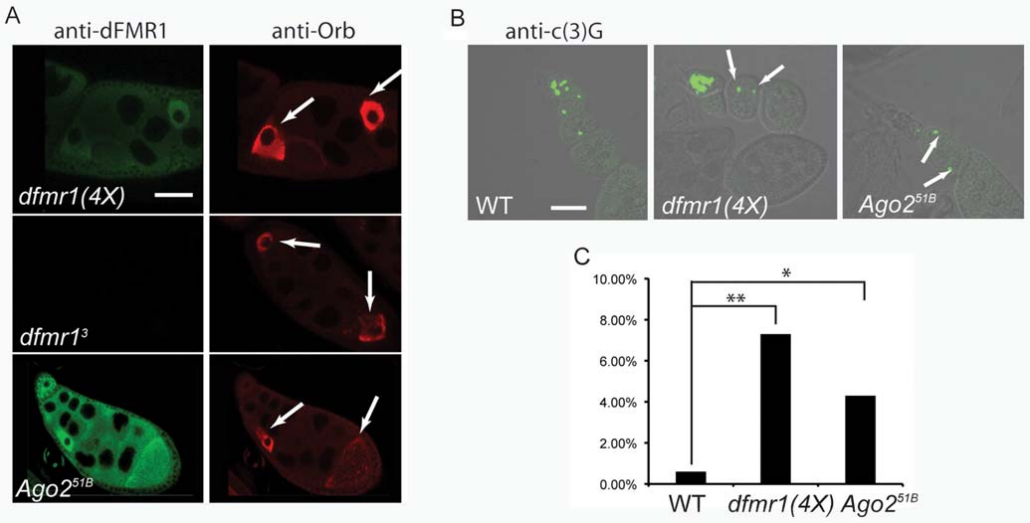
Loss of Ago2 results in a developmentally abnormal oocyte phenotype. (A) A two-oocyte mis-specification defect (marked with arrows) in a single late-stage egg chamber as observed by ectopic expression of Orb. Whole-mount ovaries labeled for dFMR1 (green) and Orb (red) from *dfmr1(4X)*, *dfmr1^3^*, and *Ago2^51B^* flies. Scale bar is 100 µm. (B) A two-oocyte mis-specification defect in a single early egg chamber as observed by c(3)G expression. A single synaptonemal complex is present in a WT egg chamber, whereas egg chambers from *dfmr1(4X)* and *Ago2^51B^* flies display two synaptonemal complexes (marked with arrows). Scale bar is 50 µm. (C) Penetrance of two-oocyte mis-specification defect. Percentage of egg chambers displaying the oocyte defect is shown on the y-axis as visualized by counting stage 6–10 egg chambers marked with anti-c(3)G staining of WT, *dfmr1(4X)*, and *Ago2^51B^* ovaries. WT (n = 155), *dfmr1(4X)* (n = 122), *Ago2^51B^* (n = 137). *P<0.05 and **P<0.005 using a one-sided Fisher exact test.

Egg chambers from *dfmr1^3^* null flies display multiple oogenesis defects at a low penetrance, including two oocytes mis-specified in a single egg chamber [40]. Notably, *dfmr1(4X)* over-expression flies also displayed the rarely seen phenotype of two oocytes mis-specified in a single egg chamber (instead of a single oocyte in a single egg chamber), using the cytoplasmic polyadenylation element binding protein homologue, Orb, as an oocyte marker (Figure 4A). Although there were no gross morphological mutant phenotypes observable in the majority of *Ago2* null egg chambers, we did observe that the loss of Ago2 resulted in the two oocytes mis-specified in a single egg chamber defect at a low penetrance (Figure 4A). To quantify this phenotype we carried out ovary staining with the synaptonemal complex component marker c(3)G in order to see early mis-specification of two oocytes [41]. We found that *dfmr1(4X)* flies displayed this phenotype at 7.3% penetrance (n = 122) (Figure 4B, C). We observed the defect at 4.3% (n = 137) in the *Ago2* null egg chambers (P<0.05) in comparison to a penetrance of 0.6% (n = 155) in the wild-type egg chambers (Figure 4C). Further genetic epistasis analyses based on the double oocyte phenotype were not possible because both gain and loss of dFMR1 expression caused the same defects in oogenesis.

### Ago2 regulates *dfmr1* transcript levels during oogenesis

**Figure 5:**
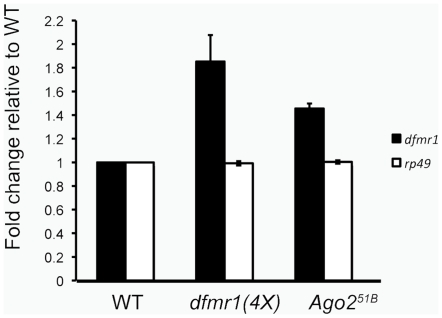
Ago2 regulates *dfmr1* mRNA. *dfmr1* and *rp49* transcript levels were measured in four biological replicates using quantitative real time PCR (QT-PCR) from ovary cDNA from WT, *dfmr1(4X)*, and *Ago2^51B^* flies. QT-PCR for *dfmr1* was normalized to *rp49*. QT-PCR for *rp49* was normalized to *28S*. Data are graphed as mean fold change relative to WT ± s.e.m.

To determine how Ago2 regulates dFMR1 expression, we analyzed how loss of Ago2 affects *dfmr1* steady-state transcript levels in adult ovaries using quantitative real-time PCR (QT-PCR) and Northern analyses (Figures 5 and S2). Because we observed dFMR1 up-regulation in *Ago2* null egg chambers, we conducted QT-PCR and Northern analyses in RNA lysates from ovaries in which we could easily isolate mass amounts of tissue materials. We detected an average of 1.5 fold increase in *dfmr1* transcript levels from *Ago2* null ovaries in comparison to *dfmr1* transcript levels from wild-type ovaries (Figures 5 and S2). The difference between the fold up-regulation of *dfmr1* mRNA (∼1.5×) compared with the maximum fold up-regulation of dFMR1 protein levels (2–4×) in the *Ago2* null strain suggests that *dfmr1* translational efficiency may be affected by the loss of Ago2. To test whether Ago2 induces a general down-regulation of transcript levels during oogenesis, QT-PCR was used to detect the levels of another transcript, *rp49*, which codes for ribosomal protein RpL32. No change in *rp49* mRNA levels was observed in the *Ago2* null background (Figure 5).

### Is the *dfmr1* transcript a target of the endogenous siRNA pathway?

We next wanted to determine whether the *dfmr1* transcript was being directly regulated through the canonical endogenous siRNA pathway that uses processed long dsRNA to target and degrade mRNAs. The *mir-34* locus is less than 400 bp downstream of *dfmr1* and is transcribed in the opposite direction relative to *dfmr1*. Transcription of both genes could provide a potential source of dsRNA that could serve as a substrate for the siRISC. We failed to detect specific small RNAs from the *dfmr1* region using Northern analyses and no significant matches to the *dfmr1* locus were detected from deep sequencing results from small RNAs isolated from ovaries, heads and S2 cell lines (data not shown and the generous sharing of unpublished data and personal communication from G. Hannon and P. Zamore). These data suggest that *dfmr1* is not likely a direct target of the canonical endogenous siRNA pathway.

### Is Ago2 cleavage activity required for *dfmr1* regulation?

Based on the data above, which suggests that *dfmr1* is not a direct target of the endogenous siRNA pathway, we questioned whether Ago2 might play a unique role in regulating *dfmr1* that does not require Ago2 cleavage activity. To answer this question, we examined how *dfmr1* was regulated in another *Ago2* mutant fly-strain, *Ago2^V966M^*. The *Ago2^V966M^* flies carry a point mutation that reduces the cleavage activity of Ago2 by eight-fold, but does not affect the expression levels of Ago2 [42]. If Ago2-dependent catalysis of the *dfmr1* transcript or any other transcript were required for regulation of *dfmr1*, then we would expect to see an *Ago2* null-like larval NMJ phenotype in the *Ago2^V966M^* larval NMJs in comparison to wild-type larval NMJs. Unlike the NMJ analyses of *Ago2* null larvae, the bouton and branching numbers from *Ago2^V966M^* larval NMJs were similar to wild-type NMJs (Figure 1). Additionally, dFMR1 was not up-regulated in *Ago2^V966M^* mutant egg chambers (Figure S3), suggesting that Ago2 suppresses dFMR1 expression through a mechanism that bypasses a functional Ago2 catalytic domain.

## Discussion

Dosage of Fragile X expression must be tightly regulated to prevent the deleterious effects seen in either loss- or gain-of-function models observed in mice, *Drosophila*, and most importantly human patients. To our knowledge, three regulators of the *Drosophila* Fragile X protein had been previously reported [24], [43], [44]. Here, we provide genetic evidence that dFMR1 is also regulated by Ago2 in the nervous system and in the germ line. This finding is exciting and novel in that to our knowledge Ago2 has not been previously shown to regulate the protein levels of an endogenous target in the nervous system and germ line and few other endogenous protein targets have been identified [34].

It has become clear in the last decade that mechanisms of translational control are fundamental for proper synaptic function related to learning and memory [45]. Furthermore, components of the RISC pathway have also been shown to localize to the synapse in *Drosophila* where they are involved in translational regulation, and to the peripheral nerve axons in mammals [46], [47]. Our findings, along with the aforementioned, support the conclusion that Ago2, in addition to other RISC pathway components are active where rapid translation in response to cell signaling is required.

Because siRNA pathway mutants are not lethal like their micro RNA (miRNA) counterpart mutants, and do not exhibit gross morphological defects, a potential regulatory role for the siRNA pathway during development has been overshadowed. We have shown that loss of Ago2, a core member of the siRNA pathway, causes specific and significant defects in neurogenesis and oogenesis. The neurological defects we observed in *Ago2* mutants require dFMR1, suggesting that the role of Ago2 in neuronal development might also include additional dFMR1 specific and/or novel functions. Defects in synaptic architecture that are caused by loss of or elevation of dFMR1 levels have functional consequences at the glutamatergic NMJ synapse [27]. Zhang et al. observed that loss of dFMR1 results in elevated evoked synaptic transmission whereas pre-synaptic dFMR1 over-expression results in elevated spontaneous vesicle fusion[27]. We are interested in determining whether *Ago2* mutants might also exhibit elevated spontaneous vesicle fusion affecting *Drosophila* learning and memory.

In the studies reported here, we focused on the biological implications and regulation of one disease-related target, *dfmr1*, by the siRNA pathway component Ago2. Several endogenous siRISC targets have been identified using global analyses such as micro-arrays and deep sequencing of small RNAs [48], [49], [50], [51], [52]. However, few Ago2 targets have been characterized with respect to how mis-regulating such targets might impact the development of *Ago2* mutant animals. In *Drosophila*, the neuronal ion channel *pickpocket* (*ppk*) has been shown to be up-regulated in animals mutant for *Ago2* [29]. We noted similarities between the regulation of *dfmr1* and *ppk* by Ago2, perhaps suggesting a shared regulatory mechanism as well. Both *ppk* and *dfmr1* expression were elevated to similar levels due to loss of Ago2 [29]. The differential increase between dFMR1 protein and mRNA levels in an *Ago2* null animal suggests that Ago2 activity results in post-transcriptional regulation of *dfmr1*. We hypothesize that Ago2 might regulate its targets in a protein complex that acts to regulate the stability and/or translational efficiency of the mRNA. However, we have not yet ruled out that Ago2 might also regulate gene expression at the protein level by altering the stability or overall activity of the protein. The specific post-transcriptional mechanisms that Ago2 utilizes to regulate gene expression remain unknown for Ago2 targets such as *dfmr1* and *ppk*.

We also examined previous studies of Ago2 to understand how Ago2 might regulate *dfmr1* with diminished ribonuclease cleavage activity. Ago2 is required for normal formation of processing bodies or P bodies (ribonucleoprotein aggregates containing enzymes involved in mRNA decay and miRNA associated translational regulation) in S2 cells [53]–[55]. Ago2 has been shown to localize to neuronal granules in primary cell culture of *Drosophila* larval central nervous system (CNS) cells [56]. Additionally, Ago2 has also been shown to protect poly-A tail length of a target transgene in S2 cells [57]. All of these associated properties of Ago2 have not been shown to require a siRNA intermediate, and may help to elucidate a general mechanism for how Ago2 regulates targets such as *dfmr1*. Whether Ago2 might be acting in a complex with other siRNA pathway members or the dFMR1 protein itself to regulate *dfmr1* expression also remains to be investigated.

## Materials and Methods

### *Drosophila* stocks

The following *Drosophila* stocks were used: WT (*w^1118^*), *dfmr1(4X)* (*w^1118^; P{WTR-dfmr1}*)[20], *dfmr1^3^ (dfmr1^3^/Tm6C,Tb,Sb)* [20], *Ago2^51B^* (*Ago2^51B^/Tm3,Sb,GFP*)[29], *Ago2^414^ and P{Ago2^414^}; Ago2^414^* [35], *Ago2^V966M^* [42], and *Ago2^51B^,dfmr1^3^/Tm3,Sb,GFP* (made by standard recombination procedures and verified by genomic PCR).

### Immunohistochemistry

Brains were dissected in 1X PBS and fixed in 4% paraformaldehyde (PFA)/PBST (1X PBS +0.2% Triton-X 100), for 20 minutes at RT. Brains were washed in PBST, blocked in 5% Normal Goat Serum (NGS) for 1 hour at 4°C and incubated in primary antibody overnight at 4°C. Brains were washed and incubated in secondary antibody for 2 hours at RT. Brains were mounted in Mounting Medium (KPL 71-00-16). Ovaries were stained in the same manner as described for the brains except that they were dissected in 1X Robbs (55 mM sodium acetate, 40 mM potassium acetate, 100 mM sucrose, 10 mM glucose, 1.2 mM MgCl_2_, 1 mM CaCl_2_, 100 mM HEPES [pH 7.5]) and fixed in 4%PFA/PBS. Ovary staining with anti-c(3)G was done as described previously [41]. Primary antibodies used were: anti-dFMR1 (6A15) 1∶1000 [15], anti-Orb (6H4) 1∶30 (Developmental Studies Hybridoma Bank, Iowa), anti-DE-cadherin (DCAD2) 1∶20 (DSHB, Iowa) and anti-c(3)G 1∶500, a kind gift from Scott Hawley [41]. Secondary antibodies used were: FITC-conjugated goat anti-mouse IgG1 1∶500 (Southern Biotech), Texas Red-conjugated goat anti-mouse IgG2a 1∶500 (Southern Biotech), and Cy5-conjugated donkey anti-rat 1∶350 (Jackson ImmunoResearch).

### Immunohistochemistry of larval NMJ

Preparations were fixed and stained as previously reported with the following modifications [58], [59]: third instar wandering larvae were dissected along the dorsal midline in Ca^2+^ saline, pinned flat onto dishes coated in Sylgard (Dow-Corning), and fixed for either 45 minutes (anti-CSP staining at 1∶200) [60] or 25 minutes (Texas Red-conjugated HRP staining at 1∶200 (Jackson ImmunoResearch)) in 4% PFA/PBS. All anti-CSP DAB stained preparations used to quantify synaptic boutons were mounted in Cytoseal XYL mounting medium (Richard-Allan Scientific). Fluorescent preparations used to visualize gross synaptic morphology were mounted in Vectashield mounting medium (Vector laboratories). A stack of images was taken to capture a 2-dimensional image through the entire depth of the NMJ, and presented in Figure 1A as a maximum projection for each genotype. Anti-CSP staining was visualized using a Vectastain ABC Elite Kit with NiCl_2_ enhancement and images were quantified at 1000X using a Leica DME microscope. A Leica TCS SP confocal microscope using software version 2.6.1 was used to capture the maximum projection from multiple sections of Texas Red-conjugated-HRP images at 600X.

### NMJ morphological analysis

Quantification of the NMJ morphology in larvae was done as described with the following modifications [58], [59]. Anti-CSP stained type I boutons at the muscle 6/7 NMJ of abdominal hemisegment 3 were quantified in *w^1118^*, *dfmr1^3^*, *dfmr1(4X), Ago2^414^*, *P{Ago2};Ago2^414^, Ago2^51B^* and double mutant *Ago2^51B^, dfmr1^3^* larvae. The number of boutons was averaged for all larval hemisegments counted (n≥13) from each genetic background. Branch numbers were counted as previously described [59]. Statistical analyses were performed using one-way ANOVA followed by Dunnett's test.

### Microscopy

All confocal images were taken with the Leica TCS SP confocal microscope using software version 2.6.1. The experiments shown in Figure 2 were completed as a single set, as defined below. The experiments shown in Figure 3 were completed as a single set. The experiments shown in Figure S3 were completed as a single set. A single set of images is defined as follows: for each set, images for all genotypes were taken at the same time, with the same documented settings, including pixel size, resolution, dimensions, hardware parameters, laser and scanner settings. Figures 1 and 4 (which were not quantitative images) were assembled with images taken at different photomultiplier tube settings. Quantification for Figures 2, 3 and S3 were calculated using the Leica TCS SP quantification software. For Figures 3 and S3, one line of identical length was drawn through one focal plane of each image (average of 4 scans) for each set. Leica quantification software plots the pixel intensity (0–255) versus position along that line (µm) for each channel acquired (anti-dFMR1 and anti-DE-cadherin using sequential scanning). This process was carried out on multiple egg chambers (n≥10) and repeated in multiple staining experiments. A representative image was chosen for the figures. For Figure 2, due to the non-uniform staining of dFMR1 in the brain, the mean fluorescent intensities within three defined regions of interest (ROI) (same areas and dimensions of ROIs kept for all images) were averaged. This process was carried out in multiple brains (n>5) from each genetic background. The mean intensity of the background staining observed in *dfmr1* null flies was subtracted from the mean fluorescent intensity measured in each ROI of each genetic background before normalization to WT to calculate the fold change.

### Quantitative RT-PCR

Ovary pairs from 3–6 day old females were dissected in 1X Robbs buffer. Total RNA from ovaries was extracted using TRI Reagent (Ambion) and bromochloropropane (BCP) for RNA extraction, and was further purified using columns from RNeasy Mini Kit (Qiagen) and on-column treatment with Qiagen DNase I. 0.5 µg RNA was used to generate cDNA using random hexamers (Invitrogen Superscript III kit). Real-time PCR was carried out using SYBR GreenER qPCR superMix Universal (Invitrogen) and the Mx3005P PCR system (Stratagene). We carried out four biological replicates with four separate RNA pools and four separate reverse transcription reactions for ovary experiments. Subsequently, each biological replicate was run in triplicate technical replicates for QT-PCR analyses. Cycling program was as follows: 95°C for 10 minutes, 40 cycles of 95°C for 30 seconds, 60°C for 1 minute, 72°C for 30 seconds followed by a melting curve analysis. Analyses were carried out using median cycle threshold (C_T_) values and normalization to *rp49* or *28S* as internal controls. Sequences for QT-PCR primers: 5′ TGGTCAATGGCACGTCCTAA (forward) and 5′ TTCTAGCCATCTGTGAGCTGTTG (reverse) for *dfmr1*. Primer sequences for *rp49* and *28S* were as described [61].

### Northern Analysis

Total RNA from ovaries was isolated using the RNeasy Mini Kit with Qiashredder columns (Qiagen). Northern analysis was performed using the NorthernMax-Gly kit (Ambion) as described by the manufacturer's instructions with the following modifications. 25 µg total RNA was run per lane. Ambion Bright-Star membranes pre-hybridized in ULTRAhyb (Ambion) for 30 minutes at 68°C, and probed overnight with ^32^P-labeled *dfmr1* fragment or ^32^P-labeled β-tubulin fragment, which served as a loading control. Probes were synthesized and removed using the StripAble RNA probe synthesis and removal kit (Ambion). Sequences of primers used to generate Northern probes 5′ AAGAAGCCCAGAAGGATGGT (forward) and 5′ T7 + TTCTCCTCCAGCTCGATGTT (reverse) for *dfmr1* and 5′ CTGGAGCGCATCAATGTGTA (forward) and 5′ T7 + TGTGTGAGTTGGAAGCCTTG (reverse) for *β-tubulin*. RNA levels were assessed using phosphorimaging techniques and Image Quant software (version 2.4).

### Western Analysis

Ovaries from 3 day-old females fed on grape plates or heads were dissected in 1X Robbs buffer. Extracts were prepared using extraction buffer (20 mM HEPES (pH 7.5), 100 mM KCl, 5% glycerol, 100 µM Na3VO4, 10 mM EDTA, 0.1% Triton-X, 1 mM DTT, Complete Protease Inhibitor (Roche)) and mixed with NuPage LDS 4X sample buffer (Invitrogen). Samples were boiled for 10 minutes, separated on a 4–12% Bis-Tris gel (Invitrogen), and transferred to a PVDF membrane (Immobilon-P, Millipore). To detect dFMR1, membranes were incubated with anti-dfmr1 antibody (5A11) 1∶2000 (DSHB, Iowa). To detect the loading controls, actin and β-tubulin, membranes were incubated with anti-actin (JLA20) 1∶2000 (DSHB) or anti-β-tubulin (E7) 1∶2000 (DSHB).

## Supporting Information

Figure S1 dFMR1 protein levels are increased in heads and ovaries from ago2 mutant flies. (A) Western analysis of head extracts from: WT, dfmr13, dfmr1(4X), Ago2414 and Ago251B. Anti-dFMR1 (5A11) was used to visualize dFMR1 levels. β-tubulin was used as a loading control. (B) Quantification of dFMR1 levels from head lysates was carried out with two biological replicates (except for Ago2414 (carried out once) using Image Quant software. The average relative levels of dFMR1 are represented as the ratio of dFMR1 to β-tubulin and were normalized to WT. Data are graphed as mean + s.d. (C) Western analysis of ovary lysates from: WT, dfmr1(4X), Ago251B, and Ago2414. Actin was used as a loading control. (D) Graphical representation of the quantification of the western analyses in ovary lysates. Quantification was carried out with two or more biological replicates as in (B) except that Actin was used for normalization. Data are graphed as mean + s.d. (Note that in panels A and C irrelevant lanes were removed to simplify the presentation of the data. All of the samples shown in each panel are derived from the same blot.)
(0.74 MB TIF)

Figure S2 dfmr1 transcript levels are elevated in Ago2 null ovaries. (A) Representative Northern blot for dfmr1 transcript levels. Total ovary RNA samples were probed for dfmr1 transcripts (top panel) and β-tubulin (lower panel) to provide a loading control. (B) dfmr1 RNA from WT, dfmr1(4X), and Ago251B ovary lysates were quantified and averaged from 2 blots using Image Quant software. The average levels of the dfmr1 transcript are represented as the ratio of dfmr1 levels to β-tubulin levels and are normalized to transcript levels from WT tissue. Data are graphed as mean ± s.e.m.
(0.32 MB TIF)

Figure S3 Ago2 does not require robust endoribonuclease activity to regulate dFMR1 during oogenesis. Representative images of whole-mount ovaries labeled for dFMR1 (green) from WT and Ago2V966M flies. Scale bar is 26.65 µm. The graphs plot the pixel intensity (0 to 255 as quantified by Leica software) versus position (µm) along a line drawn through the egg chamber (n>10 for each genetic background).
(0.26 MB TIF)
