# Supplementary figures and images for: Argonaute2 Suppresses Drosophila Fragile X Expression Preventing Neurogenesis and Oogenesis Defects
Source: PLoS One. 2009 Oct 27;4(10):e7618. doi: 10.1371/journal.pone.0007618 (PMC2770736; doi:10.1371/journal.pone.0007618)

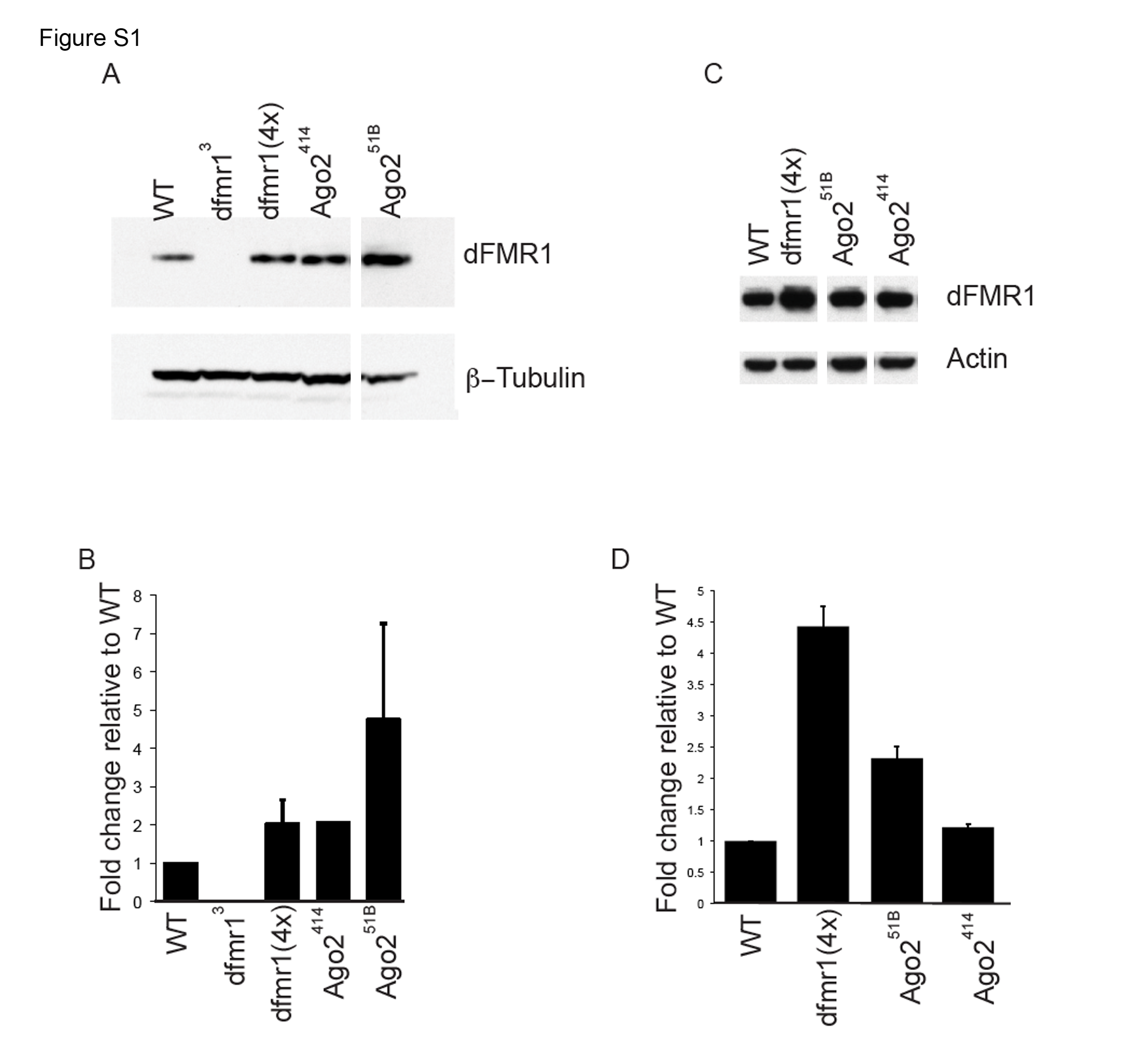

Supplement: Figure S1 — dFMR1 protein levels are increased in heads and ovaries from ago2 mutant flies. (A) Western analysis of head extracts from: WT, dfmr13, dfmr1(4X), Ago2414 and Ago251B. Anti-dFMR1 (5A11) was used to visualize dFMR1 levels. β-tubulin was used as a loading control. (B) Quantification of dFMR1 levels from head lysates was carried out with two biological replicates (except for Ago2414 (carried out once) using Image Quant software. The average relative levels of dFMR1 are represented as the ratio of dFMR1 to β-tubulin and were normalized to WT. Data are graphed as mean + s.d. (C) Western analysis of ovary lysates from: WT, dfmr1(4X), Ago251B, and Ago2414. Actin was used as a loading control. (D) Graphical representation of the quantification of the western analyses in ovary lysates. Quantification was carried out with two or more biological replicates as in (B) except that Actin was used for normalization. Data are graphed as mean + s.d. (Note that in panels A and C irrelevant lanes were removed to simplify the presentation of the data. All of the samples shown in each panel are derived from the same blot.) (0.74 MB TIF) [file pone.0007618.s001.tif]

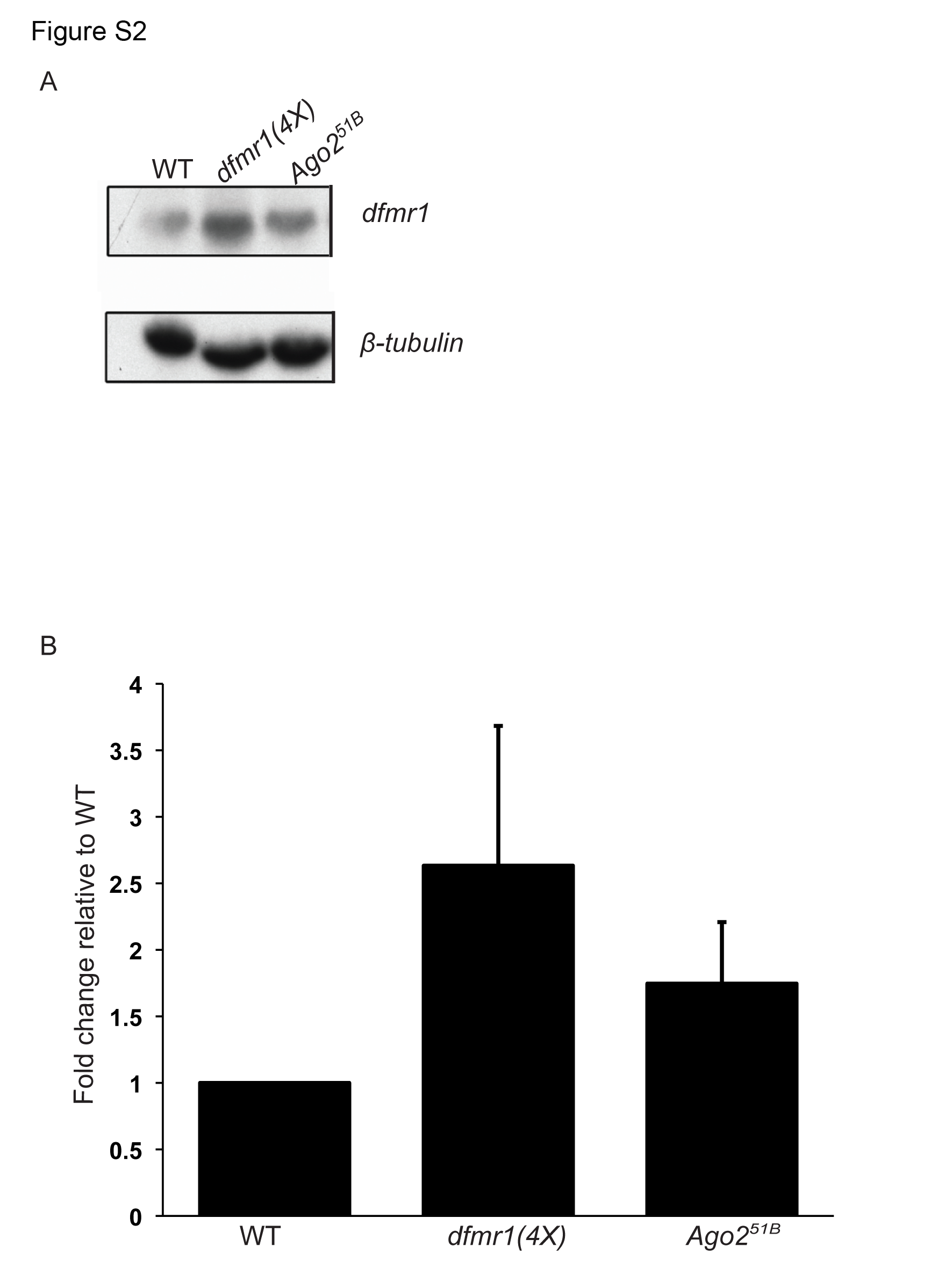

Supplement: Figure S2 — dfmr1 transcript levels are elevated in Ago2 null ovaries. (A) Representative Northern blot for dfmr1 transcript levels. Total ovary RNA samples were probed for dfmr1 transcripts (top panel) and β-tubulin (lower panel) to provide a loading control. (B) dfmr1 RNA from WT, dfmr1(4X), and Ago251B ovary lysates were quantified and averaged from 2 blots using Image Quant software. The average levels of the dfmr1 transcript are represented as the ratio of dfmr1 levels to β-tubulin levels and are normalized to transcript levels from WT tissue. Data are graphed as mean ± s.e.m. (0.32 MB TIF) [file pone.0007618.s002.tif]

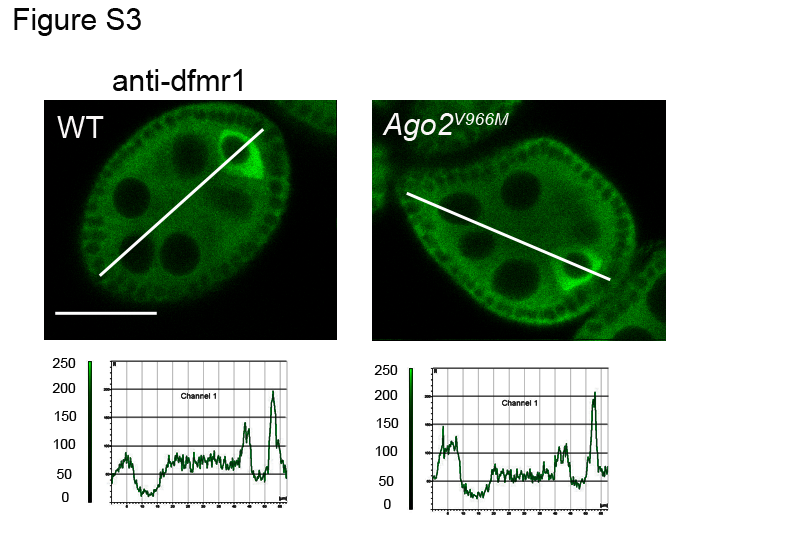

Supplement: Figure S3 — Ago2 does not require robust endoribonuclease activity to regulate dFMR1 during oogenesis. Representative images of whole-mount ovaries labeled for dFMR1 (green) from WT and Ago2V966M flies. Scale bar is 26.65 µm. The graphs plot the pixel intensity (0 to 255 as quantified by Leica software) versus position (µm) along a line drawn through the egg chamber (n>10 for each genetic background). (0.26 MB TIF) [file pone.0007618.s003.tif]
